# A rare case report of renal clear cell carcinoma with multiple skin metastases and a review of the literature

**DOI:** 10.3389/fonc.2024.1461791

**Published:** 2024-12-04

**Authors:** Wei Wang, Yongcun Kang, Xiaona Qu, Yang Li, Hongyan Zhou

**Affiliations:** ^1^ Department of Ultrasound, The Second Affiliated Hospital of Dalian Medical University, Dalian, China; ^2^ Department of Radiology, The Second Affiliated Hospital of Dalian Medical University, Dalian, China; ^3^ Department of Oncology, The Second Affiliated Hospital of Dalian Medical University, Dalian, China

**Keywords:** skin metastasis, renal cell carcinoma, ultrasound, survival, prognosis

## Abstract

Renal cell carcinoma is the most common type of primary renal cancer, and clear cell carcinoma is the most common subtype, accounting for approximately 70% of all adult renal cell carcinoma cases. At the time of diagnosis, many patients already have metastatic manifestations. Cutaneous metastasis of renal clear cell carcinoma is very rare and usually represents a poor prognosis, mostly affecting the head and neck. In this paper, we report a case of renal clear cell carcinoma with multiple cutaneous metastases, including a chest wall mass for more than 10 years and an abdominal wall mass for 1 year. A 69-year-old man with a history of diabetes mellitus was admitted to the hospital for examination of a right chest and abdominal wall mass and peripheral pain, and ultrasonography suggested a solid space-occupying lesion in the left kidney, which was considered malignant, and a solid mass in the right chest and abdominal wall, which was considered metastatic. A subsequent abdominal CT scan showed malignant tumors in the left kidney and adrenal region, and multiple metastatic tumors in the liver, pancreas, right thoracoabdominal wall, and the abdomen. To clarify the nature of the pathology, an ultrasound-guided puncture of the right abdominal wall mass was performed, and the pathological diagnosis was clear cell carcinoma, with immunohistochemistry suggesting a renal clear cell carcinoma origin. The patient died within 6 months.

## 1 Introduction

Cutaneous metastasis of renal clear cell carcinoma is a rare metastatic pathway and mostly occurs at the advanced stage of the disease. Such patients typically have a poor prognosis (1). Most skin metastases occur in the head and face. This article conducts a retrospective analysis of a patient with renal clear cell carcinoma accompanied by multiple skin metastases. This analysis is presented in combination with the clinical and imaging features of this case to enhance clinicians’ understanding of and diagnostic ability for such lesions.

## 2 Case presentation

**Figure 1:**
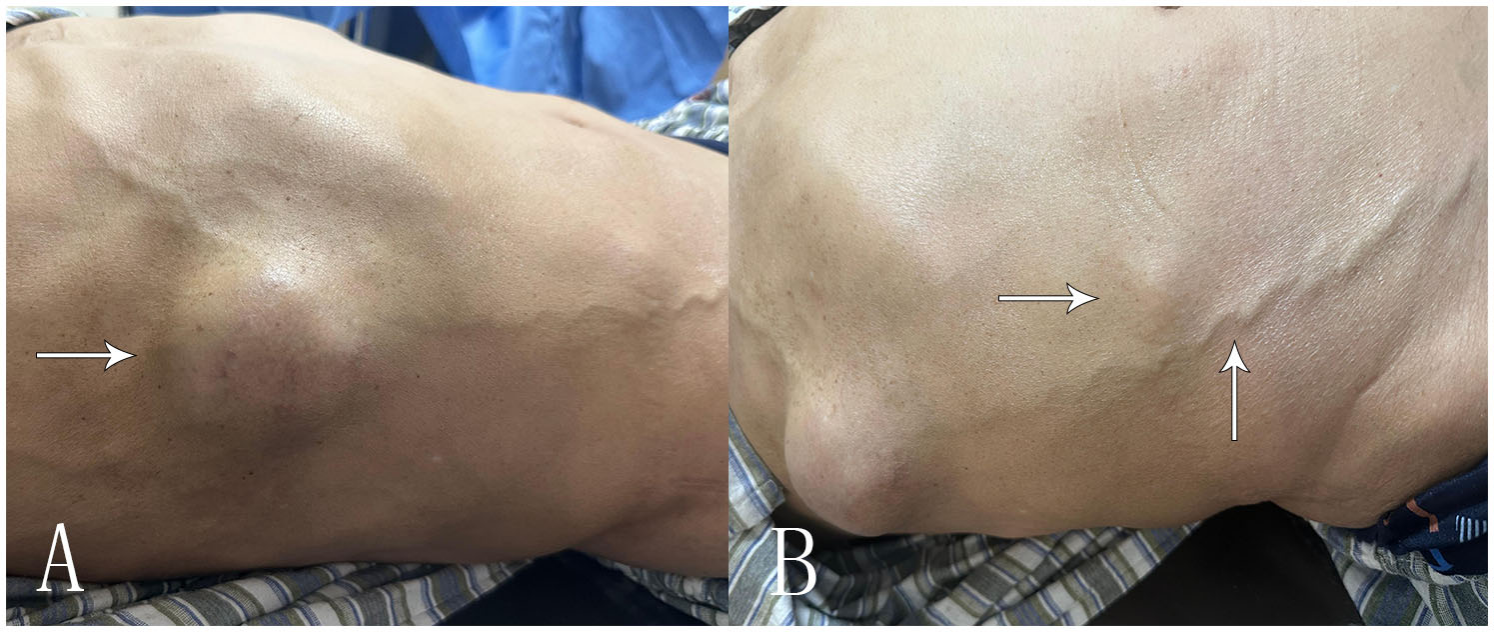
Clinical presentation of the patient’s chest wall and abdominal wall masses. **(A)** The arrow shows the right chest wall mass of the patient’s chief complaint for 10 years. **(B)** The arrow shows the smaller right abdominal wall mass with thick blood vessels visible on the skin surface.

**Figure 2:**
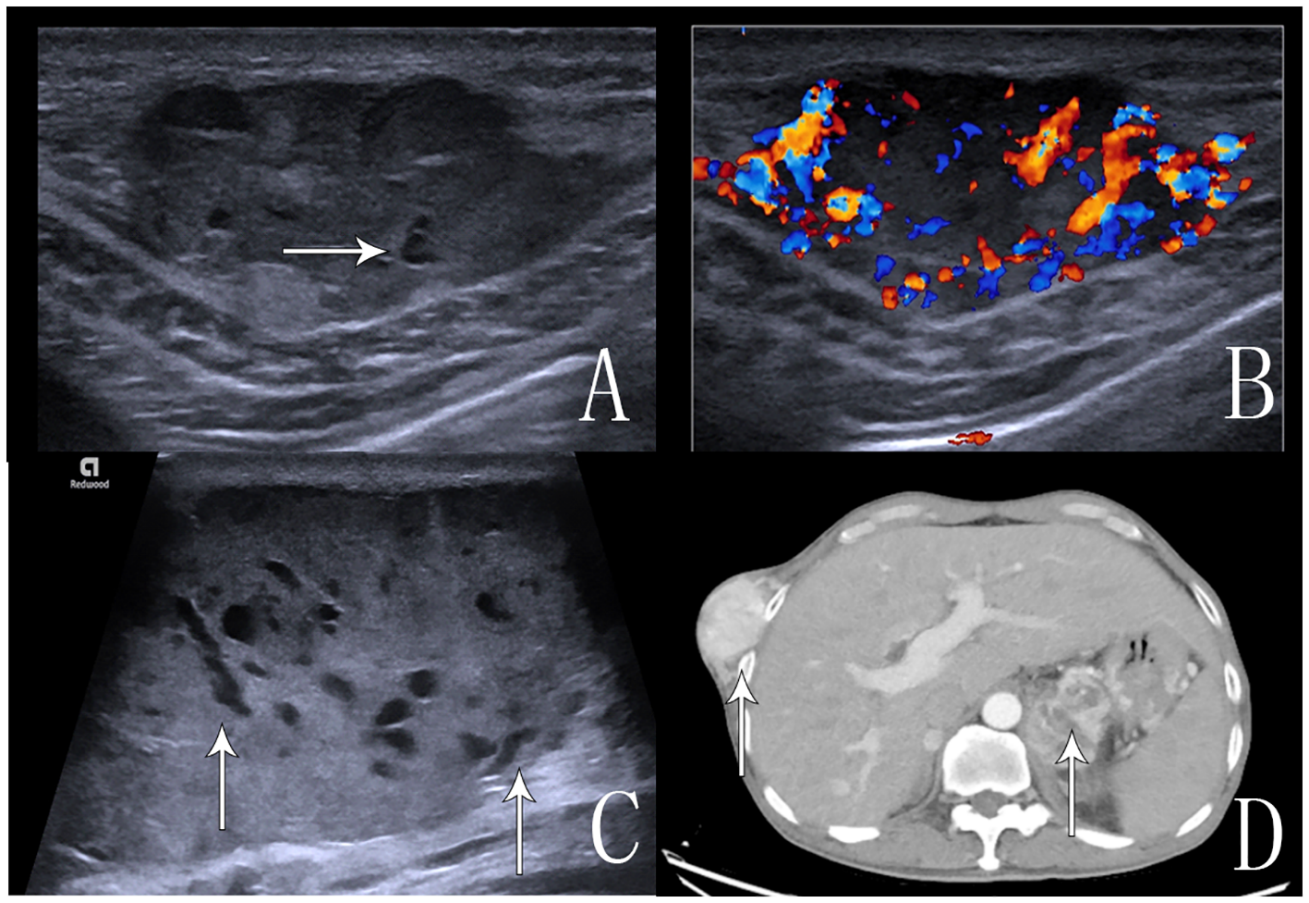
Ultrasound and CT images of the abdominal wall and chest wall masses. **(A)** An ultrasound image that shows anechoic echogenicity in the hyperechoic mass in the right abdominal wall. **(B)** An ultrasound image that shows abundant blood flow signals in the mass in CDFI. **(C)** An ultrasound image that shows multiple anechoic echoes in the hyperechoic mass in the right chest wall, which is a “fissure”-like alteration. **(D)** An enhanced CT scan, the arrow shows inhomogeneous enhancement of the right chest wall mass and left kidney tumor.

**Figure 3:**
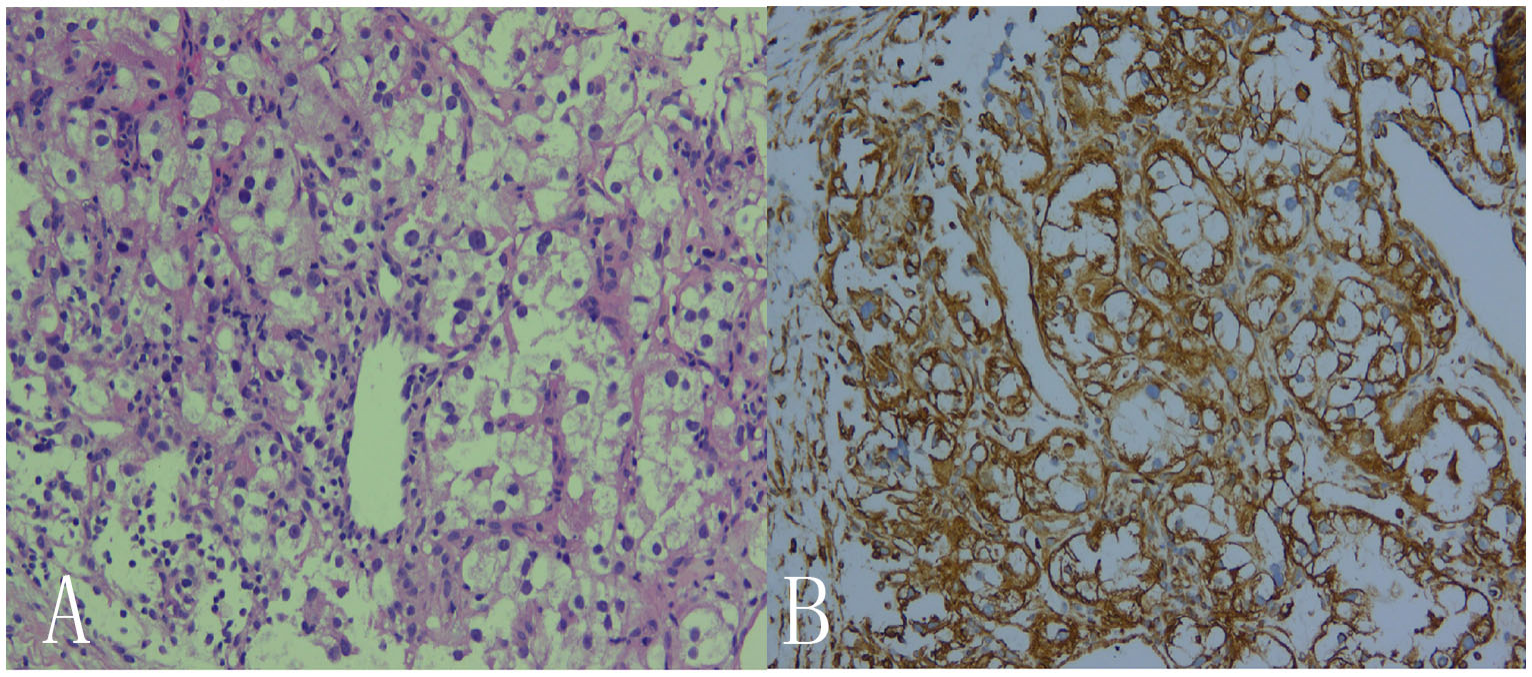
Histological observation showed that, microscopically, the cancer cells were seen to be arranged in solids and were hyaline or granular. Small thin-walled sinusoidal vessels consisting of reticular intervals were scattered in the tumor tissue. (**A**: HE×200, **B**: IHC×200).

A 69-year-old male patient was admitted to the hospital for examination due to a mass in the right thoracoabdominal wall and peripheral pain. He was previously healthy, with no family history of genetic disease, a history of smoking for 10 years, and a history of diabetes mellitus for more than 10 years. Furthermore, a mass had been found on the right side of the chest wall more than 10 years previously and an abdominal wall mass 1 year previously. A physical examination showed a raised mass in the right sternoabdominal wall (**Figure 1A**), which was consistent with the color of the skin, normal skin temperature, and a soft texture, and dilated blood vessels were visible on the surface of the skin around the mass in a tortuous pattern (**Figure 1B**). The following laboratory tests were conducted: creatinine 42.4 μmol/L (58-110 μmol/L), carbohydrate antigen125 was 68.8 U/ml (0-24.00 U/ml), and syphilis spirochete antibody 383S/CO (≤1.2S/CO). Ultrasonography showed two hypoechoic echoes on the right side of the thoracic and abdominal wall, measuring 4*6 cm and 3*1.5 cm, respectively, with clear borders and regular morphology. A large number of fissure-like echoes were seen inside, the probe was slightly deformed after pressure, and a large number of blood flow signals were seen inside the fissures (**Figures 2A–C**). The upper pole of the left kidney showed a slightly high echo with a size of 14.3*9.4cm, an unclear boundary, and an irregular shape, and the internal echo was uneven, and the blood flow signal was visible in CDFI. The solid occupational lesion in the left kidney was considered malignant; the right chest and abdominal wall solid mass was considered metastatic. A CT enhancement scan of the left kidney and adrenal region showed a class round mixed low-density shadow with a size of approximately 9 * 5cm and the enhancement was unevenly strengthened. The liver and spleen were shown to be hyperechoic with the enhancement unevenly strengthened and multiple hypodense shadows were found in the right chest, abdominal wall, and abdominal cavity with the enhancement unevenly strengthened (**Figure 2D**). Malignant tumors in the left kidney and adrenal region and multiple metastases in the liver, pancreas, right chest, abdominal wall, and abdominal cavity were considered. An ultrasound-guided puncture biopsy was performed on the right thoracic and abdominal wall mass, and the pathologic results indicated clear cell carcinoma. The immunohistochemistry results were as follows: CAIX(+), CD10(+), vimentin(+), RCC(partially +), CK7 (-), CD117(-), and Melan-A(-), which suggested an origin of renal clear cell carcinoma (**Figure 3**). The patient did not undergo radiotherapy and only took analgesic drugs such as morphine hydrochloride tablets and amino tramadol tablets. He died within 6 months of follow-up.

## 3 Discussion

Renal cell carcinoma accounts for 80-90% of primary renal malignancies in adults, and based on histology and molecular subtypes, the most common type of RCC is clear cell carcinoma, which occurs frequently due to mutations in the VHL gene (2). Renal cell carcinoma is deeply located, clinical symptoms are sometimes not obvious, and metastasis has occurred in approximately 1/3 of patients at the time of diagnosis. Metastasis is most common in the lungs, bones, liver, and brain, and skin metastasis is very rare, only seen in 2.8%-6.8% of patients (3). Most of these are scalp metastases (4, 5) and a few of them are metastases in the face and neck with metastases in the chest and abdominal wall being even rarer.

From 2000 to now, a total of 29 cases of skin metastasis of renal clear cell carcinoma have been reported (**Table 1**), of which 5 cases were female and the remaining 24 cases were male. These included 22 cases in the head and neck (5, 6, 19, 22–40), 3 cases in the limbs (20, 21, 36), 3 cases in the trunk (15, 17, 41), and 1 case in the scrotum (42). Most of the cases showed painless lumps, and only 2 cases had painful symptoms (29, 33). The lumps were mostly red or blue-purple, and a few were flesh-colored, and could be accompanied by bleeding. Among the 29 cases,10 cases showed a skin manifestation as the first sign, reminding us to pay attention to skin manifestations, which is sometimes an important clue in the diagnosis of renal clear cell carcinoma. The occurrence of skin metastases after diagnosis can occur up to 19 years after surgery (6), which suggests the importance of long-term follow-up after renal clear cell carcinoma.

**Table 1:** Summary of reported cutaneous metastases of clear cell carcinoma of the kidney.

Location	Gender	Age	Appearance of mass	Time of mass appearance	Reference
Head and neck	Male	55	Unknown	Postoperation	(22)
	Male	75	Red violet nodule	7 years after surgery	(23)
	Male	77	Red violet nodule	12 years after diagnosis	(24)
	Male	72	Red violet nodule	3 years after surgery	(25)
	Male	66	Red violet nodule	Pre-diagnosis	(26)
	Male	65	bleeding mass	10 years after surgery	(28)
	Female	72	erythematous nodules	8 years after surgery	(5)
	Female	48	bleeding mass	Pre-diagnosis	(29)
	Male	65	erythematous nodules	Pre-diagnosis	(30)
	Female	83	Red violet nodule	19 years after surgery	(6)
	Male	52	Red violet nodule	Pre-diagnosis	(31)
	Male	39	bleeding mass	2.3 years after surgery	(32)
	Male	86	Red violet nodule	Pre-diagnosis	(33)
	Male	70	Unknown	Pre-diagnosis	(34)
	male	51	Unknown	11 years after surgery	(19)
	Male	66	Red violet nodule	0.6 years after surgery	(35)
	Male	84	Unknown	9 years after surgery	(36)
	Male	40	Red violet nodule	Pre-diagnosis	(37)
	Male	63	bleeding mass	Pre-diagnosis	(38)
	Male	73	bluish-red nodule	10 years after surgery	(39)
	Male	64	erythematous nodules	After diagnosis	(40)
	Female	69	Red violet nodule	6 years after surgery	(27)
Limb	male	63	bleeding mass	0.6 years after diagnosis	(20)
	Male	66	Unknown	After diagnosis	(36)
	Male	64	bleeding mass	3 years after diagnosis	(21)
Trunk	Male	49	erythematous nodules	Pre-diagnosis	(17)
	Female	68	Unknown	11 years after diagnosis	(41)
	Male	59	bleeding mass	10 years after surgery	(15)
Scrotum	Male	56	bleeding mass	Pre-diagnosis	(42)

The most common route of distant organ metastasis of renal cell carcinoma is lymphovascular metastasis, through the thoracic duct, up into the head and neck, and the other mode of dissemination is the hematogenous route through the paravertebral venous plexus, which drains blood from multiple arteriovenous fistulas formed during tumorigenic angiogenesis, both of which can explain the occurrence of unusual sites of metastasis, particularly in the head, neck, and trunk (7, 8). Metastases may even exist before the primary renal tumor is detected. It has been suggested that cutaneous metastasis is common in the chest and abdomen due to the proximity of the anatomical site to the kidneys (9). However, in this case, the original lesion was clear cell carcinoma of the left kidney, the metastatic site was the right thoracic wall and the right neck, and large blood vessels were visible around the mass. The metastasis method should first consider the distant metastasis pathways of kidney cancer.

Clinically, cutaneous metastases typically present as painless and non-bleeding smaller masses that are flesh-colored, purplish, or blue. The appearance may be confused with hemangioma, pyogenic granuloma, Kaposi’s sarcoma, infected cutaneous cysts, or cutaneous lymphoma; therefore, we should consider these lesions in the differential diagnosis (10). It is the asymptomatic nature of skin metastasis that makes it easy to be overlooked by patients, which leads to a prolonged diagnosis of the disease. Studies have shown that >90% of patients with skin metastases from renal cancer are male and tend to be of the clear cell subtype.

On ultrasound images, skin metastasis of renal clear cell carcinoma manifests as a substantial mass with clear boundaries, which is usually accompanied by abundant blood flow signals, and such thick blood vessels may suggest skin metastasis of visceral tumors (11). Therefore, attention should be paid to skin tumors with abundant blood flow during ultrasound examination, which can be combined with a medical history and clinical manifestations.

The typical histology of renal clear cell carcinoma shows a nested, tubular, or vesicular growth pattern, consisting of cells with clear cytoplasm and the presence of a complex network of blood vessels, basically, capillaries around each nest of tumor cells, which is a diagnostic indication of renal clear cell carcinoma (12).

Cutaneous metastases are usually considered to be an advanced manifestation of the disease with a poor prognosis (13). As shown in this case, when the skin metastases are detected, other body organs may be involved, resulting in tumor-specific survival of usually less than 6 months. In a study by Rikard Ohlsson et al., it was found that 96% of patients died within 36 months of skin metastasis (14). The patient in this case was found to have had a thorax and abdominal wall mass for more than 10 years without tenderness or skin color change which was not treated by medical treatment. There were no clinical manifestations of hematuria in this examination, and the survival time was longer than that of conventional patients with skin metastasis, which was quite different from the survival and prognosis of previous studies. In previous cases, the survival time after skin metastasis was 108 months (15). Combined with the disease condition and treatment plan, it is believed that the patient’s prolonged survival was caused by the drug intervention, and the survival of the patient, in this case, was more than 10 years without any treatment, which may be related to the dormancy mechanism of tumor cells (16). The initial metastatic cells did not obtain sufficient vasogenesis or isolated cells could not initiate cell division, leading to the dormancy of metastatic tumor cells. Studies have also shown that cell dormancy is due to the canceling of the proliferation of cancer cells by other mechanisms, which affects the growth of tumor cells (17). The cause of this mechanism is still unclear, and it is related to other abnormal indicators in patients or individual differences. A large number of cases need to be included in a study to explore what factors lead to tumor cell dormancy and whether it is related to anti-vascular mechanisms and immunotherapy, which will provide great benefits for the treatment of tumors.

Recurrent metastasis occurs in 20% to 30% of renal cancer patients after surgery so the postoperative totalized management plan is of great significance in the era of precision medicine. The latest consensus (18) suggests that prognostic stratification according to the International mRCC Database Consortium (IMDC) should be used to determine the follow-up treatment plan of patients, which can effectively improve the prognosis of patients and reduce recurrence. We could also monitor patients with different grades according to the prognostic stratification, not only for long-term follow-up of thoracic and abdominal organs but also to be alert for abnormal skin masses in high-risk patients.

Previous skin metastases were mostly found in postoperative patients on follow-up, and in this case, the patient complained of pain and a chest and abdominal wall mass, with skin metastasis as the first symptom (19), which also reminds us that we should not only be alert to postoperative patients on follow-up in our clinical work but also be sensitive to this kind of painless skin mass growth which is sometimes the first clue to diagnose tumor disease.

## 4 Conclusion

Skin metastasis of renal cell carcinoma is rare and is usually found during postoperative follow-ups, sometimes as the first symptom of undiscovered tumor disease (20). Among the patients followed up after surgery, the average occurrence time is 4-5 years, and some skin involvement appears at long intervals, the longest being more than 19 years, which suggests that long-term follow-up is very important, and this atypical and asymptomatic skin manifestation can easily be misdiagnosed as benign diseases such as hemangiomas and rashes. Therefore, ultrasonographic diagnosis of skin masses in this group of patients is very important. Combining the ultrasound characteristics of this case with the literature review, we hope to improve physicians’ understanding of the ultrasound manifestations of this type of lesion, and since the prognosis of patients with skin metastases is poor, early diagnosis and further guidance of treatment are important to improve the survival and prognosis of these patients.

## Data Availability

The original contributions presented in the study are included in the article/supplementary material. Further inquiries can be directed to the corresponding authors.
